# Inspections of radiocesium concentration levels in rice from Fukushima Prefecture after the Fukushima Dai-ichi Nuclear Power Plant accident

**DOI:** 10.1038/srep08653

**Published:** 2015-03-03

**Authors:** Naoto Nihei, Keitaro Tanoi, Tomoko M. Nakanishi

**Affiliations:** 1 Graduate School of Agricultural and Life Sciences, The University of Tokyo, 1-1-1, Yayoi, Bunkyo-ku, Tokyo 113-8657, Japan

## Abstract

We summarize the inspections of radiocesium concentration levels in rice produced in Fukushima Prefecture, Japan, for 3 years from the nuclear accident in 2011. In 2011, three types of verifications, preliminary survey, main inspection, and emergency survey, revealed that rice with radiocesium concentration levels over 500 Bq/kg (the provisional regulation level until March 2012 in Japan) was identified in the areas north and west of the Fukushima nuclear power plant. The internal exposure of an average adult eating rice grown in the area north of the nuclear plant was estimated as 0.05 mSv/year. In 2012, Fukushima Prefecture authorities decided to investigate the radiocesium concentration levels in all rice using custom-made belt conveyor testers. Notably, rice with radiocesium concentration levels over 100 Bq/kg (the new standard since April 2012 in Japan) were detected in only 71 and 28 bags out of the total 10,338,000 in 2012 and 11,001,000 in 2013, respectively. We considered that there were almost no rice exceeding 100 Bq/kg produced in Fukushima Prefecture after 3 years from the nuclear accident, and the safety of Fukushima's rice were ensured because of the investigation of all rice.

The Great East Japan Earthquake occurred on March 11, 2011, and was immediately followed by the accident at the Fukushima Dai-ichi Nuclear Power Plant (NPP) of Tokyo Electric Power Company (hereafter referred to as the nuclear accident). Radioactive substances, such as cesium^1^,^2^,^3^, strontium^4^, plutonium^5^,^6^, and the others^7^, emitted from the nuclear accident settled onto agricultural land in Fukushima Prefecture, as well as on land in neighboring prefectures^8^,^9^,^10^, causing contamination of soil and agricultural products. To fulfill the requirements of the Food Sanitation Act in Japan (Law No. 233 issued in 1947), on March 17, 2011, the Ministry of Health, Labor and Welfare (MHLW) established a provisional regulation level of 500 Bq/kg for radiocesium in cereals, vegetables, meat, and fishery products. In April 1, 2012, a new maximum limit of 100 Bq/kg was established as a new standard of radiocesium in general food excluded infant food, milk, water and beverages^11^,^12^. To revitalize agriculture within the prefecture, Fukushima Prefecture has been promoting the decontamination of agricultural land while implementing radioactive substance absorption suppression measures for agricultural products^13^. To verify the safety of agricultural and fishery products, the Nuclear Emergency Response Headquarters have been conducting emergency environmental radiation monitoring of agricultural and fishery products (hereafter referred to as monitoring inspections) as part of the emergency response in accordance with the special measure of the Nuclear Disaster Act in Japan. Targeted items and sampling locations for the monitoring inspections are determined by discussions with municipalities, consideration of the amount of production, and value of any shipment. By March 2013, the total number of item types was approximately 450, equaling approximately 81,500 samples. When the results of a monitoring inspection indicate that the level of radiocesium exceeds 500 Bq/kg (the provisional regulation level, from March 17 in 2011 until March 31 in 2012) or 100 Bq/kg (the new standard, after April 1 in 2012), the relevant municipalities are requested to restrict shipments, based on instructions issued by the Director-General of the Nuclear Emergency Response Headquarters in Japan. There are some reports of the contamination of the agricultural products immediately after the nuclear accident^3^,^14^,^15^.

Rice is the main staple food of the Japanese diet, and it is the most valuable agricultural product in Fukushima Prefecture. Hence, Fukushima Prefecture and the Central Government of Japan have issued more detailed directives for the inspection of rice within the prefecture compared to other agricultural products (Table 1). In 2011, as a precaution, the Central Government of Japan requested suspension of rice planting, based on a special measure of the Nuclear Disaster Act, for the relevant municipal governments that were in the controlled areas within a 20-km radius of the Fukushima Dai-ichi NPP, and where radiocesium exceeding 5,000 Bq/kg was detected in the soil. In addition, monitoring inspections are conducted on three samples in each municipality for each item; however, for rice, the number of samples for inspection is significantly higher, and even more detailed inspections are performed in areas where measured values exceed the detection limits in 2011. In 2012, as a precaution, rice planting was suspended in areas that produced rice with radiocesium levels exceeding 500 Bq/kg in 2011 and in areas where evacuation orders had been issued. In addition to these measures, Fukushima Prefecture implemented enhanced inspections of all rice produced in the prefecture (named as inspection of all rice in all rice bags^16^, hereafter referred to as inspection of all rice). For this purpose, manufacturers have developed and produced equipment that can efficiently inspect all rice in Fukushima. Moreover, prefectures and municipalities have compiled information on individual farm households and built inspection frameworks. The results of these inspections are publicly announced first in newspapers and then on Fukushima Prefecture webpage^17^,^18^,^19^ and Fukushima-no Megumi Anzen Taisaku Kyogikai webpage^16^. In 2013, the inspection of all rice was adopted as a regular monitoring procedure as part of the emergency response in accordance with the special measure of the Nuclear Disaster Act in Japan.

Based on these results, this study analyzes the radioactive content in rice in the three-year period after the nuclear accident, and it also evaluates the performance of the equipment used to inspect all rice.

## Methods

### Inspections in 2011

After the nuclear accident in 2011, a preliminary survey before harvest was conducted to understand the trends in radioactive content in rice. In the preliminary survey, when the radiocesium concentration levels in the agricultural land were 1,000 Bq/kg in 15-cm depth average or lower, one sample was surveyed in each former municipalities (classifications as of February 1, 1950, including 374 sectors of the Prefecture) within the municipalities. However, in the case of the number of former municipalities was low and the number of survey locations in the municipality was less than five, the number of samples surveyed was set to five in municipalities. In the other municipality, the number of samples surveyed was set to five in municipalities. Thus, in this manner, a total of 441 samples were analysed. The sampling was performed about one week before harvesting. Unpolished rice was collected by reaping from the standing crop and then threshed, dried, and processed. The inspection method involved filling 100-ml containers with rice and taking measurements for 2,000-s using a germanium semiconductor detector at the Fukushima Agricultural Technology Centre (conducted in accordance with the “Manual for Measuring Radioactivity of Foods in Cases of Emergency” published by the MHLW in Japan^20^). The detection limit was approximately 10 Bq/kg, and the results have been publicly announced on Fukushima Prefecture website^17^.

For the main inspection, samples were taken from unpolished rice that was harvested, dried, and processed within areas subject to the surveys. These areas were those presumed to have high levels of radiocesium (based on the radiation dosage distribution map published by the Ministry of Education, Culture, Sports, Science and Technology^10^). Two samples were collected for every 15 hectares (generally one sample unit in every settlement) in municipalities where the radiocesium concentration levels exceeded 200 Bq/kg in the preliminary survey, and two samples were collected in every former municipality in other survey sectors. In cases where the number of samples surveyed was fewer than five in any given municipality, the minimum number was set to five samples; thus, a total of 1,174 samples were measured. The inspection methods were similar to those used in the preliminary survey, and the results were publicly announced on the Fukushima Prefecture website^18^.

An emergency survey after the main inspection was undertaken by Fukushima Prefecture itself to reassure the public about food safety. The inspections were conducted on one or more samples per farm household in areas where measurements during the main inspection or preliminary survey exceeded detection limit values, 10 Bq/kg. Sampling was performed in a total of 23,247 farm households, using NaI or other types of scintillation counters. The survey methods and results have been publicly announced on the Fukushima Prefecture website^19^.

### Inspections in 2012 and 2013

**Figure 1:**
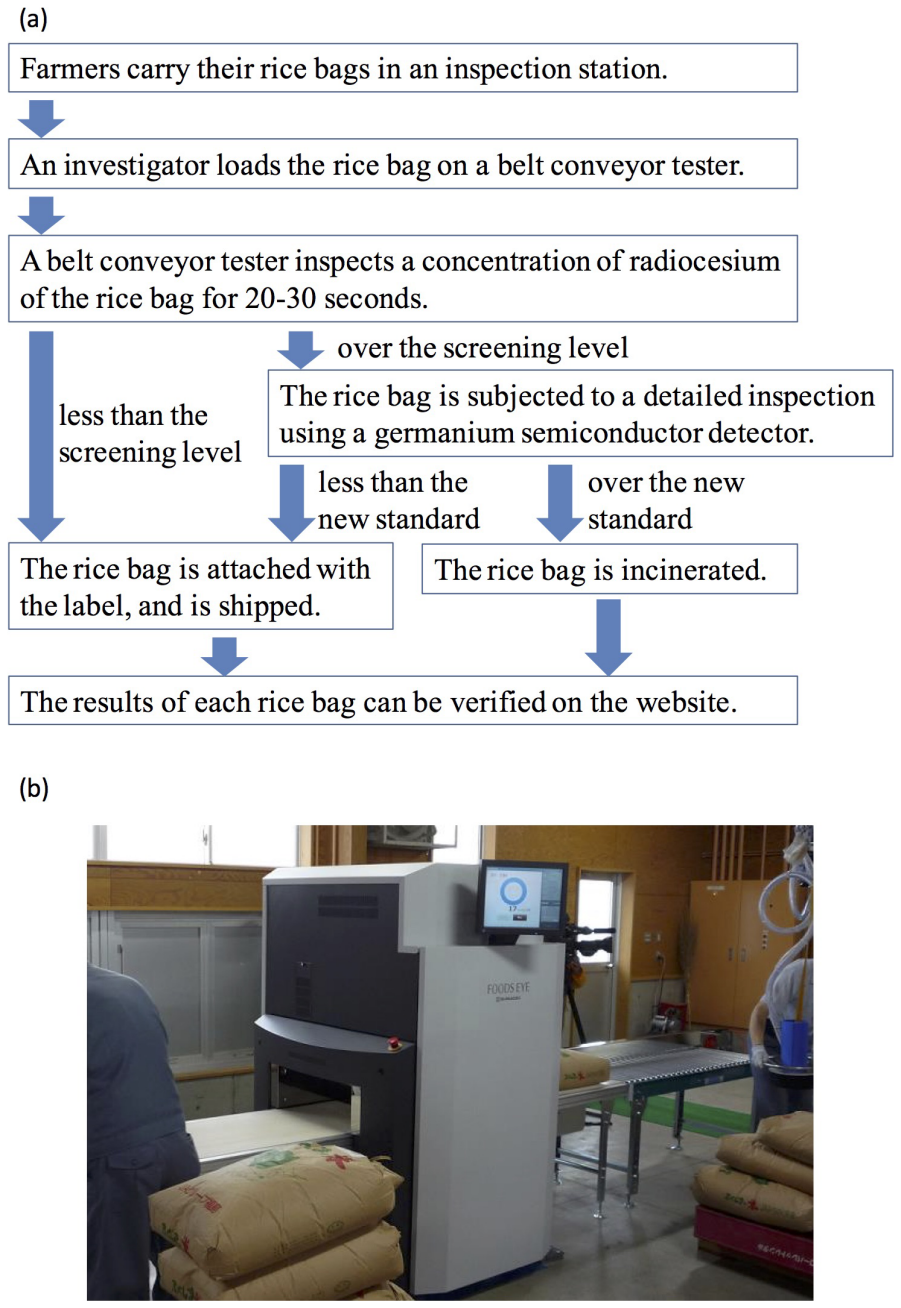
Overview of the inspection of all rice performed from 2012. (a) Scheme of the inspection of all rice. The weight of the rice bags are uniformly 30 kg. The screening level was 50–85 Bq/kg, which was dependent on the belt conveyor testers and the background level. (b) A picture of one of the belt conveyor testers. There are 5 belt conveyor testers certified by Fukushima Prefecture to perform the inspection of all rice.

The inspections conducted by Fukushima Prefecture in 2012 targeted all the rice produced within Fukushima Prefecture (approximately 360,000 tons) to reassure the public about food safety. Because there were limitations on the number of germanium semiconductor detectors available, and monitoring inspections would have taken considerable time, manufacturers were requested to develop a belt-conveyor-type radiocesium concentration tester (hereafter referred to as the belt conveyor tester) for taking measurements. The belt conveyor testers were equipped with NaI or other types of scintillation counters, and the entire measurement section was shielded by lead or iron. Rice bags weighing 30 kg passed along the belt at a rate of two or three rice bags per minute and were examined to ensure whether radiocesium concentration level exceeded 100 Bq/kg, which was stipulated by the Food Sanitation Act^12^. This measurement method was conducted according to the “Screening Method for Radioactive Cesium in Food Products”, as indicated by the MHLW^21^, which stipulates that the value of each screening level calculated using individual equipment must be half or more of the standard value (100 Bq/kg). Fukushima Prefecture installed approximately 200 belt conveyor testers in various areas throughout the prefecture, and inspections were performed to coincide with shipments from producers (by the end of July 2013, approximately 10,338,000 samples had been inspected). The scheme for the inspection of all rice is shown in Figure 1, and can be described as follows: 1) farmers carry their rice bags to an inspection station, 2) their rice bags are sealed with a bar code label that includes the farmer's information, 3) the investigators load the rice bag on a belt conveyor tester and upload the farmer's information with a bar code reader, and 4) the belt conveyor tester measures the radiocesium concentration levels from each rice bag for 20–30 s. If the inspection result is less than the screening level, a label bearing an individual identification number is attached to the rice bag indicating the bag was inspected, and then the bag is shipped. If the inspection result exceeds the screening level, the bag is further subjected to a more detailed inspection using a germanium semiconductor detector, and it is isolated and stored until the measurement value is finalized. Finally, the result for each rice bag and the results for the rice produced from each area are posted on Fukushima-no Megumi Anzen Taisaku Kyogikai website^16^.

### Compilation of Data

**Figure 2:**
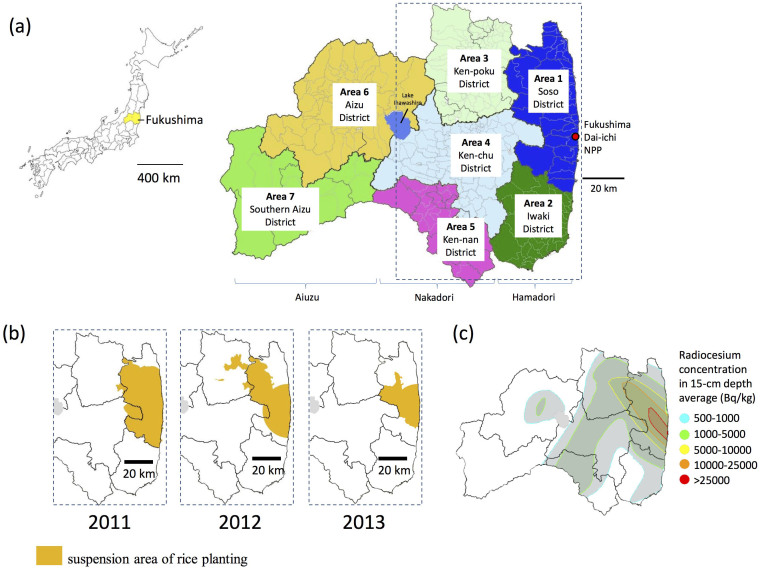
Seven administrative sectors of Fukushima Prefecture and suspension area of rice planting after the nuclear accident. (a) Seven administrative sectors of Fukushima Prefecture; Area 1 is 30–50 km to the NPP (Soso District), Area 2 is 30–50 km to the south of the NPP (Iwaki District), Area 3 is approximately 30–80 km to the northwest of the NPP (Ken-poku District), Area 4 is approximately 20–70 km west of the NPP (Ken-chu District), Area 5 is approximately 40–80 km to the southwest of the NPP (Ken-nan District), Area 6 is approximately 70–130 km to the west of the NPP (Aizu District), and Area 7 is between 100 and 150 km west of the NPP (Southern Aizu District). Area 1, 2 are called Hamadori (Coastal region); Area 3, 4, and 5 are called Nakadori (Central region); and Area 5, 6 are called Aizu. NPP: nuclear power plant. (b) suspension area of rice planting after the nuclear accident. (c) The Radiocesium concentration of soil in 15-cm depth average (Bq/kg), which was made from the data from the Ministry of Agriculture, Forestry and Fisheries of Japan (http://www.s.affrc.go.jp/docs/map/pdf/02_1_bunpu_zeniki.pdf: in Japanese). The blank paper map of Japan was provided by Fukushima prefecture, which was made from the data source of National Land numerical information (XML format) in Geospatial Information Authority of Japan website (http://nlftp.mlit.go.jp/ksj/jpgis/datalist/KsjTmplt-N03.html). After the blank paper map was scanned, the data was modified by using GIMP2.8 (http://www.gimp.org).

Data from the municipalities and the former municipalities within Fukushima Prefecture were compiled by classifying the municipalities according to the radiocesium concentration levels in rice: under 25 Bq/kg, 25–100 Bq/kg, 100–500 Bq/kg, and over 500 Bq/kg. For compiling the results, areas were classified into seven administrative sectors according to their distance from the nuclear power plant (Figure 2): Area 1 is 30–50 km to the north of the nuclear power plant (Soso District), Area 2 is 30–50 km to the south of the plant (Iwaki District), Area 3 is approximately 30–80 km to the northwest of the plant (Ken-poku District), Area 4 is approximately 20–70 km west of the plant (Ken-chu District), Area 5 is approximately 40–80 km to the southwest of the plant (Ken-nan District), Area 6 is approximately 70–130 km to the west of the plant (Aizu District), and Area 7 is approximately 100–150 km west of the plant (Southern Aizu District). Areas 1 and 2 are called Hamadori (Coastal region); Areas 3, 4, and 5 are called Nakadori (Central region); and Areas 5 and 6 are called Aizu.

## Results

### Inspection results for 2011

**Figure 3:**
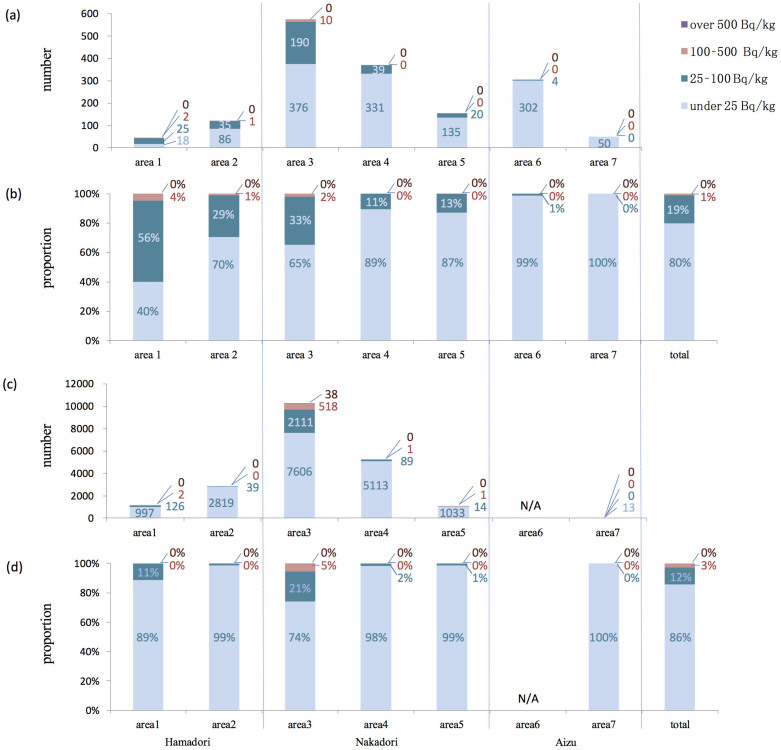
Inspections of radiocesium in rice conducted in 2011. (a) Total number of the preliminary surveys and the main inspections conducted in Areas 1–7. (b) The proportion from the data of (a). (c) Number of emergency surveys. (d) The proportion from the data of (c). The regions from Areas 1–7 are shown in Figure 2. The blue bar indicates levels under 25 Bq/kg, the dark blue bar 25–100 Bq/kg, the red bar 100–500 Bq/kg, and the purple bar over 500 Bq/kg. N/A: not applicable.

The results of the preliminary survey and main inspection conducted in 2011 are combined in Figures 3a and 3b. Area 1 (number of inspection = 45, hereafter referred to as n), located 30–50 km north of the nuclear power plant showed the highest contamination among the seven administrative areas. In fact, 40% of the locations had measurements of 25 Bq/kg or lower, 55.6% had measurements of 25–100 Bq/kg, and 4.4% had measurements of 100–500 Bq/kg, and the maximum value obtained was 154 Bq/kg. In Fukushima Prefecture as a whole (n = 1624), 79.9% of the locations had measurements of 25 Bq/kg or lower, 19.3% had measurements of 25–100 Bq/kg, and 0.8% had measurements of 100–500 Bq/kg, and the maximum value obtained was 500 Bq/kg.

The results of the emergency survey are shown in Figures 3c and 3d. These were primarily conducted at Areas 1–5, where numerous samples were identified having values close to or exceeding the detection limits for the preliminary survey and main inspection. The results of the emergency survey as a whole (n = 20,520) indicate that 85.7% of locations had measurements of 25 Bq/kg or lower, 11.6% had measurements of 25–100 Bq/kg, and 2.5% had measurements of 100–500 Bq/kg, and the maximum value obtained was 1,154 Bq/kg.

### Inspection results for 2012

**Figure 4:**
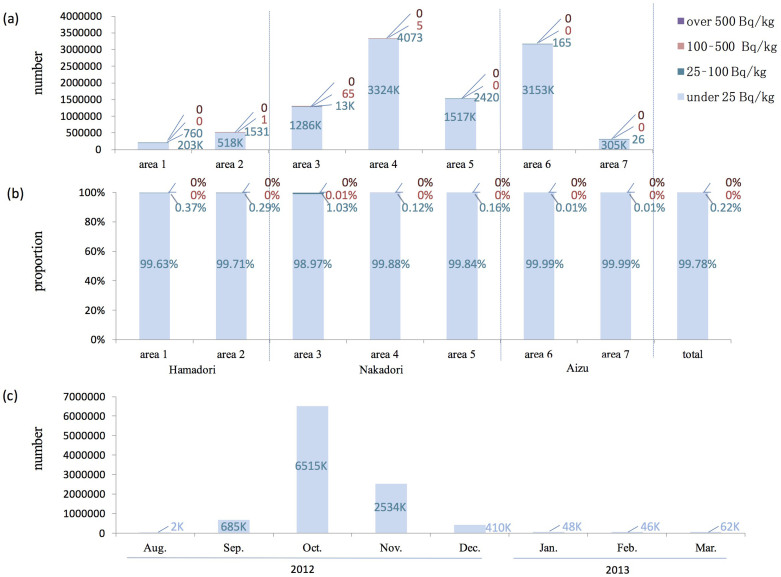
Inspections of radiocesium in rice conducted in 2012. (a) Number of inspections of rice bags. (b) The proportion from the data of (a). (c) Number of inspections of rice bags by month. The regions are shown in Figure 2. The blue bar indicates levels under 25 Bq/kg, the dark blue bar 25–100 Bq/kg, the red bar 100–500 Bq/kg, and the purple bar over 500 Bq/kg.

The results of the inspection of all rice in 2012 are shown in Figure 4. For Fukushima Prefecture as a whole (n = 10,338,291), 99.8% of the locations had measurements of 25 Bq/kg or lower, 0.2% had measurements higher than 25–100 Bq/kg, and only 71 bags (less than 0.001%) exceeded 100 Bq/kg.

**Figure 5:**
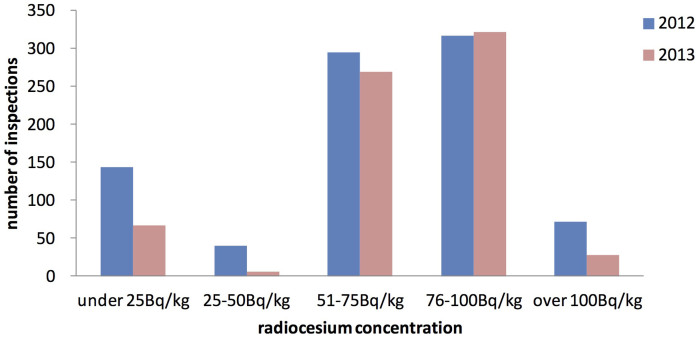
Results of detailed inspection for rice bags that exceeded the screening level by the inspection of all rice. The blue bar indicates results of detailed inspection in 2012, the red bar in 2013. 867 samples in 2012 and 692 samples in 2013 exceeded the screening levels in the inspection of all rice, and they were subjected to detailed inspection using a germanium semiconductor detector. As a result, 71 and 28 rice bags in 2012 and 2013 exceeded the new standard of radiocesium (100 Bq/kg), respectively.

In the inspection of all rice, 867 samples exceeded the screening levels and were subjected to a detailed inspection using a germanium semiconductor detector, and 8% of these 867 samples were shown to exceed 100 Bq/kg (Figure 5).

### Inspection results for 2013

**Figure 6:**
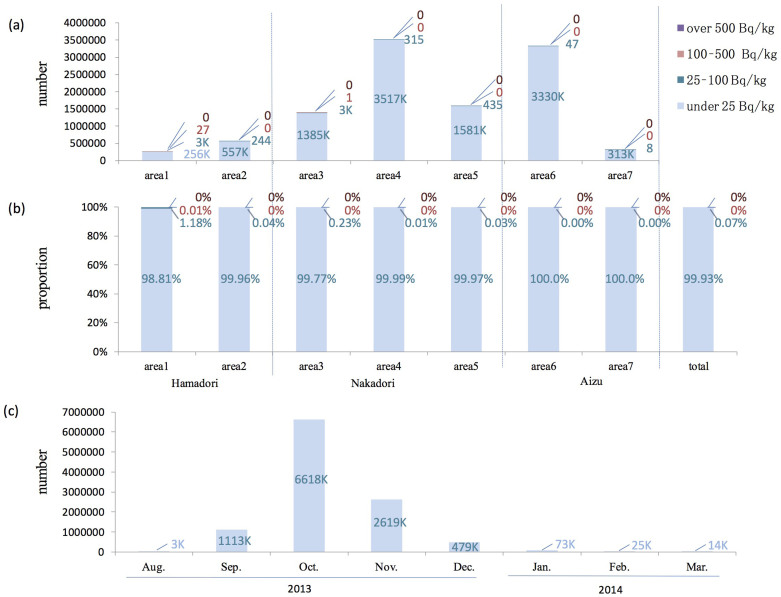
Inspections of radiocesium in rice conducted in 2013. (a) Number of inspections of rice bags. (b) The proportion from the data of (a). (c) Number of inspections of rice bags by month. The regions are shown in Figure 2. The blue bar indicates levels under 25 Bq/kg, the dark blue bar 25–100 Bq/kg, the red bar 100–500 Bq/kg, and the purple bar over 500 Bq/kg.

The results of the inspection of all rice in 2013 are similar to that in 2012 (Figure 6). Only 28 bags out of 11,001,000 exceeded 100 Bq/kg, which corresponds to 0.0003%. 99.9% of rice bags had measurements of 25 Bq/kg or lower.

692 samples exceeded the screening levels in the inspection of all rice, and 4% of these 692 samples were shown to exceed 100 Bq/kg (Figure 5).

## Discussion

**Figure 7:**
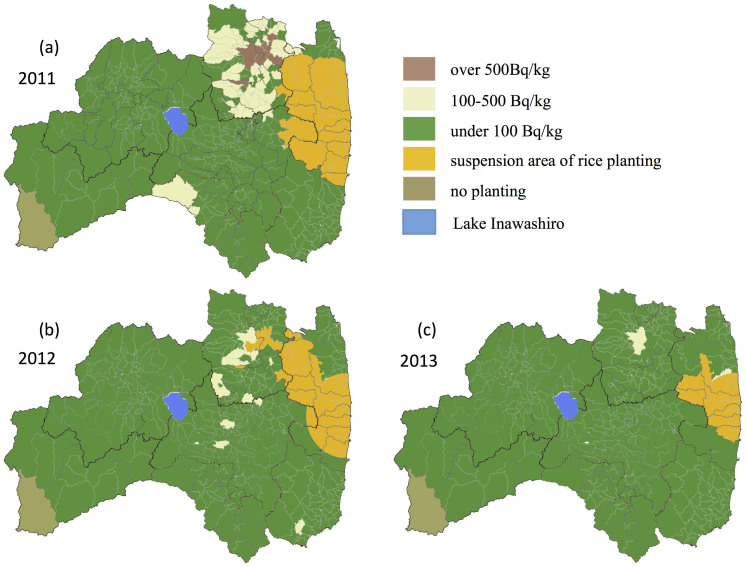
Chart sorting the former municipalities (classifications as of February 1, 1950), including 374 sectors of the Prefecture, by color according to their maximum values of radiocesium concentration levels in rice, measured by the preliminary survey, the main inspection, or the emergency survey in 2011 (a) and by detailed inspection using a germanium semiconductor detector employed after the screening of the inspection of all rice in 2012 (b) and 2013 (c). Blue area; Lake Inawashiro. The blank paper map of Japan was provided by Fukushima prefecture, which was made from the data source of National Land numerical information (XML format) in Geospatial Information Authority of Japan website (http://nlftp.mlit.go.jp/ksj/jpgis/datalist/KsjTmplt-N03.html). After the blank paper map was scanned, the data was modified by using GIMP2.8 (http://www.gimp.org).

In the preliminary survey and main inspection of 2011, 4.4% and 1.7% of rice samples had radiocesium concentration levels of 100 Bq/kg or more in Area 1 and Area 3, respectively. Figure 7a shows a chart sorting the former municipalities by color according to their maximum values in 2011, which indicates that highly contaminated rice was produced in some parts of Areas 1 and 3. These region were within 100 km northwest of the nuclear power plant, and highly contaminated by the deposited radiocesium^8^,^9^,^10^, because the plume released from the nuclear power plant from about 12 to 15 JST (Japanese Standard Time) on 15 March 2011 flowed northwestward and wet deposition with precipitation occurred in the nighttime of the same day^22^,^23^. Therefore, the proportion of rice with 100 Bq/kg or higher was greater in these areas than for other areas in 2011. However, there were many samples with 25 Bq/kg or lower in these same areas in 2011; thus, there was a range of radiocesium concentration levels within rice in a given area. Hence, the actual spread of radiocesium was heterogeneous, and the exchangeable potassium content of the soil^24^,^25^ and the soil type^26^, both of which affect the absorption rate of cesium by crops, were also heterogeneous. In addition, the radiocesium concentration levels in rice were lower in Area 2 (which was at the similar distance from the nuclear power plant as Area 1) and in Areas 4 and 5 (which were at the similar distance from the plant as Area 3) and received a lower radiocesium concentration in the agricultural land^10^. The radiocesium concentration level in the agricultural land was also low in Areas 6 and 7^10^, which were more than 100 km away from the nuclear power plant to the west. The proportion of rice with radiocesium content of 25 Bq/kg or lower in Areas 6 and 7 was 98.7% and 100%, respectively, indicating minimal impact from radiocesium. If a person ate the rice grown in Area 1 in 2011 (40% with approximately 25 Bq/kg, 55% with 25–100 Bq/kg, 4% with 100–500 Bq/kg), the calculated resulting internal exposure would be 0.05 mSv/year, assuming 60 kg annual consumption of rice^27^. These calculations assume a 1:1 concentration of ^134^Cs: ^137^Cs at the time of the nuclear accident (March 2011)^14^,^28^ and 1.9 × 10^−8^ Sv/Bq from ^134^Cs and 1.3 × 10^−8^ Sv/Bq from ^137^Cs for the deposition effective dose coefficient in oral absorption, with an average of 12.5 Bq/kg from the rice with 25 Bq/kg, 37.5 Bq/kg from the rice with 25–100 Bq/kg, and 300 Bq/kg from the rice with 100–500 Bq/kg. This is noticeably lower than the annual 1 mSv/year limit set in Japan as an internal exposure.

A comparison of the results from 2011 to 2013 reveals that 0.8% of all areas in the entire Fukushima Prefecture had contaminated rice with radiocesium concentration levels higher than 100 Bq/kg in the preliminary survey and main inspection in 2011, subsequently only 0.0007% and 0.0003% of bags had contaminated rice with over 100 Bq/kg of radiocesium in 2012 and 2013. The reason for this significant decline is assumed to be the physical reduction of radiocesium in the soil caused by factors such as decay of ^134^Cs, fixation to the soil clay, decontamination by reversal tillage, and the effects of a thorough soil improvement effort implemented to increase the exchangeable potassium content to approximately 25 mg/100 g (dry soil) or higher, which is the guideline announced by Ministry of Agriculture, Forestry and Fisheries from 2012. Moreover, planting was restricted in 2012 (by orders from the Central Government of Japan) in areas where rice in 2011 had levels exceeding 500 Bq/kg, most of which was located in Area 3, as shown in Fig 7a.

The measurement of radiocesium concentration of all rice was considered to be the very first challenge in the world. The inspections were conducted immediately after the harvesting of rice in 2012, and 99% of the entire amount was inspected during a four-month period, from September to December 2012 (Figure 4c). In October of 2012 and 2013, about 6,500,000 samples were inspected in a month, which was the peak of the inspection number. Since the inspections were conducted using approximately 200 counters, the inspection in October was performed about 1,000 samples per day by a single unit. This means that if measurements are assumed to have been taken over an 8-h period in a given day, 2 bags were inspected every minute. Considering the performance of the inspection equipment, of the samples that exceeded the screening level (867 samples), 79% also exceeded 50 Bq/kg when tested further with a germanium semiconductor detector, and 8% exceeded 100 Bq/kg (Figure 5). The proportion of samples exceeding 100 Bq/kg was extremely high in comparison with the overall rate of the inspections (0.0007%), indicating that the belt conveyor tester can be considered an effective method for screening. While the earlier versions of the manuscript were in review, the inspection revealed that no sample out of more than 10,000,000 exceeded 100 Bq/kg in 2014.

## Conclusion

Rice is the main staple food of the Japanese diet, and it is the most valuable agricultural product in Fukushima Prefecture. Therefore, after the nuclear accident at Fukushima Dai-ichi NNP, inspections were performed thoroughly for rice than for other agricultural products. Note that the proportion of rice with radiocesium concentrations exceeding 100 Bq/kg was 0.8% in 2011 and dropped to a mere 0.0007% (71 bags out of the total 10,338,000) and 0.0003% (28 bags out of the total 11,001,000) in 2012 and in 2013, respectively. In future, as agricultural operations restart in areas where planting is currently restricted, securing the safety of rice by thorough inspections and accurately communicating the inspection results will continue to be critical tools to reassure the public about food safety.

## Author Contributions

N.N. and K.T. wrote the main manuscript text and prepared figures. T.M.N. and K.T. reviewed the manuscript. N.N. was working as a Fukushima prefectural official until May, 2013, and participated in starting of the inspection of all rice.

## Figures and Tables

**Table 1:** Inspections of rice from 2011 to 2013

Year	Name	Organizer
2011	preliminary survey	Central Government of Japan^1^
2011	main inspection	Central Government of Japan^1^
2011	emergency survey	Fukushima Prefecture^2^
2012	inspection of all rice	Fukushima Prefecture^2^
2013	inspection of all rice	Central Government of Japan^1^

^1^Inspections were performed by the emergency environmental radiation monitoring on agricultural and fishery products (abbreviated as monitoring inspections) as part of the emergency response in accordance with a special measure of the Nuclear Disaster Act in Japan.

^2^Inspections were performed by Fukushima Prefecture as a self-inspection.
